# Population Regulation in Magellanic Penguins: What Determines Changes in Colony Size?

**DOI:** 10.1371/journal.pone.0119002

**Published:** 2015-03-18

**Authors:** Luciana M. Pozzi, Pablo García Borboroglu, P. Dee Boersma, Miguel A. Pascual

**Affiliations:** 1 Centro Nacional Patagónico (CONICET), Boulevard Brown 2915, 9120 Puerto Madryn, Chubut, Argentina; 2 Global Penguin Society, Seattle, Washington, United States of America; 3 Department of Biology, University of Washington, Seattle, Washington, United States of America; 4 Universidad Nacional de la Patagonia San Juan Bosco, Boulevard Brown S/N, 9120, Puerto Madryn, Chubut, Argentina; Phillip Island Nature Parks, AUSTRALIA

## Abstract

Seabirds are often studied at individual colonies, but the confounding effects of emigration and mortality processes in open populations may lead to inappropriate conclusions on the mechanisms underlying population changes. Magellanic penguin (*Spheniscus magellanicus*) colonies of variable population sizes are distributed along the Argentine coastline. In recent decades, several population and distributional changes have occurred, with some colonies declining and others newly established or increasing. We integrated data of eight colonies scattered along ∼ 600 km in Northern Patagonia (from 41°26´S, 65°01´W to 45°11´S, 66°30´W, Rio Negro and Chubut provinces) and conducted analysis in terms of their growth rates, production of young and of the dependence of those vital rates on colony age, size, and location. We contrasted population trends estimated from abundance data with those derived from population modeling to understand if observed growth rates were attainable under closed population scenarios. Population trends were inversely related to colony size, suggesting a density dependent growth pattern. All colonies located in the north—which were established during the last decades—increased at high rates, with the smallest, recently established colonies growing at the fastest rate. In central-southern Chubut, where colonies are the oldest, the largest breeding aggregations declined, but smaller colonies remained relatively stable. Results provided strong evidence that dispersal played a major role in driving local trends. Breeding success was higher in northern colonies, likely mediated by favorable oceanographic conditions. However, mean foraging distance and body condition of chicks at fledging were influenced by colony size. Recruitment of penguins in the northern area may have been triggered by a combination of density dependence, likely exacerbated by less favorable oceanographic conditions in the southern sector. Our results reaffirm the idea that individual colony trends do not provide confident indicators of population health, highlighting the need to redefine the scale for the study of population changes.

## Introduction

Determining an appropriate spatial scale, -according to the species and its life history traits-, is a critical aspect in ecological studies [1]. Metapopulation theory provides an adequate conceptual framework to understand population dynamics in spatially-structured populations, where dispersal largely influences local dynamics [2]. Seabirds provide good examples of metapopulation structure and dynamics, due to their spatial and temporal aggregation [3]. However, their colonies are often studied individually, likely because the role of dispersal is unknown for many species [4]. Increasing evidence suggests that dispersal is a key driver in local population dynamics and in the establishment of new colonies [5,6,7,8], highlighting the need for spatial scaling and management [6].

Understanding the dynamics of metapopulations largely depends on the ability to disentangle the factors that influence dispersal. Demographic processes can be influenced by density dependent or density independent factors. Farther beyond the dichotomy since the times of Nicholson [9] and Andrewartha and Birch [10], there is now increasing evidence that in vertebrate population density dependent and independent factors interact in a rather complex fashion. This interaction became particularly evident in terrestrial systems, where several studies in ungulate populations have demonstrated that carrying capacities are rather dynamic and vary depending on environmental factors (ie. rainfall). Both density dependent and density independent factors influence demographic processes in seabird populations [11]. Depending on the magnitude, the stochasticity of the environment may produce changes both via reproduction and/or survival [12]. Density independent factors may affect breeding performance either directly [13,14,15,16] or indirectly [17,18,19,20,21]. For instance, environmental variability may alter the location and/or accessibility to food sources [16,18,19,20,21]. Seabirds are central place foragers during breeding [22] that feed on areas of enhanced productivity, such as sea fronts [23]. Changes in foraging distance and in the time away from the colony alter chick feeding frequency, which reduce breeding performance either in terms of chick mortality and/or growth [17,18,19,20,21]. Changes in foraging distance may also be a consequence of density dependence [24], a hypothesis that has long been debated as an underlying mechanism of seabird population regulation. Ashmole (1963) [24] suggested that, due to enhanced intraspecific competition during the breeding season, large seabird breeding aggregations may deplete food stocks in the vicinity of their colonies, forcing birds to increase trip distances to find their prey. Several studies, including a diversity of species, have shown that seabirds breeding in larger colonies forage farther [25,26,27]. Direct evidence of this mechanism is still scarce, due to the need of measuring food abundance in the vicinity of colonies [28]. However, indirect inference, based on relationships among colony size and foraging distance, is available for several seabird species [26,27,29,30,31,32], including pursuit divers such as thick-billed murres (*Uria lomvia*) [32], Atlantic puffins (*Fratercula artica*) [29] and Adélie penguins (*Pygoscelis adeliae*) [26,27,31]. Density dependent responses may also reduce breeding performance (either in terms of breeding success or chicks growth), at larger colonies [33,34], as well as spatial segregation in foraging areas [35,36].

Beyond the specific mechanisms promoting changes in foraging distance, it is not well understood how these changes may influence the dynamics of open population. Given the influence of foraging distance in breeding performance, changes in the distance travelled may also indirectly affect recruitment. Several seabird species prospect colonies before recruitment and breeding productivity is often used as a proxy of environmental quality [37,38,39].

The Magellanic penguin (*Spheniscus magellanicus*), is the most abundant seabird breeding in Argentina [40,41], where more than 60% of the global population breeds [41,42]. Despite several studies reporting colony sizes or trends of individual colonies [15,40,41,42,43,44], no attempt has been made to understand population changes from a regional perspective. In the last decades, it became clear that while some colonies are declining [15,42] others are growing or are just being established [40,41,42,44,45]. Factors underlying the demography of Magellanic penguin populations, however, are still poorly understood. A meta-population perspective might be required to understand the dynamics of collections of penguin populations. Genetic studies from several colonies along the coastline validated this approach [46]. Specifically, what is needed is to have data and analysis of different colonies integrate in terms of colony growth rates, chick production, and the dependence of these vital rates on colony age, colony size and location. Of particular relevance is the question of the importance of dispersal and the factors that might influence, to understand the dynamics of individual colonies and the overall meta-population dynamics.

Our study examined multiple Magellanic penguin colonies from a regional viewpoint, and provided a foundation for a meta-population perspective of penguin colonies. We assessed population trends in colonies of various sizes and ages and evaluated if the observed growth rates conformed to expected growth rates in the absence of dispersal. We also evaluated breeding performance of the colonies (both in terms of breeding success and body condition of chicks at fledging) and determined foraging distance of breeding adults at sea. Finally, we integrated our data with available published information to understand what demographic processes might have driven recent population and distributional changes of Magellanic penguins in northern Argentina.

## Materials and Methods

**Fig 1:**
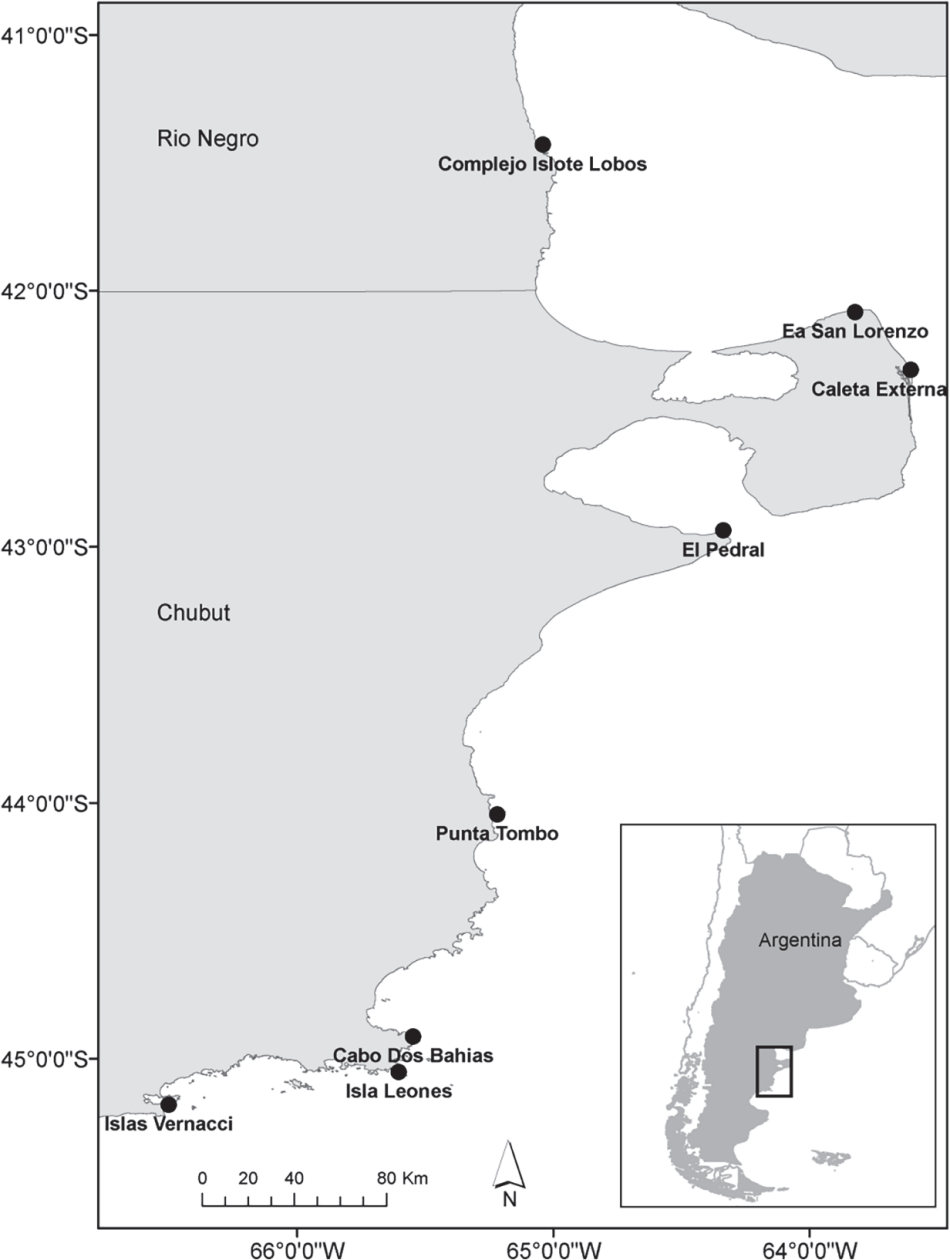
Study area. Location of the 8 Magellanic colonies studied.

Research permits for field studies were approved by Secretaría de Turismo y Áreas Protegidas (Chubut province), Dirección de Fauna y Flora Silvestre (Chubut province) and Dirección de Fauna Silvestre, Áreas Naturales Protegidas y Educación Ambiental (Río Negro province), which are the regulatory bodies for animal welfare in these provinces. Sixty-six colonies of Magellanic penguins are distributed over ∼ 2000 km of Argentine coastline in Patagonia [41,42]. Distinct oceanographic features in Patagonia determine feeding grounds [20,45,47]; therefore, different dynamics of colonies are expected between regions. Our study area spanned over 600 km in Northern Patagonia (Fig. 1), where we selected eight study colonies (or group of island colonies considered to be a single population unit) considering geographic representativeness and including the largest colonies in the area and those that were recently established. The colonies Complejo Islote Lobos (comprising islands La Pastosa and Islote Redondo), Ea. San Lorenzo (also known as La Ernestina or Punta Norte, in Península Valdés), Caleta Externa (in Península Valdés), and El Pedral are located in the Río Negro Province and north Chubut Province. Punta Tombo, Cabo Dos Bahías, Isla Leones and Islas Vernacci (which includes the islands Vernacci Este, Norte 1 and Norte 2, also referred as Bahia Bustamante) are located in the central and south Chubut Province. All colonies are located in protected areas.

### Population trends

**Fig 2:**
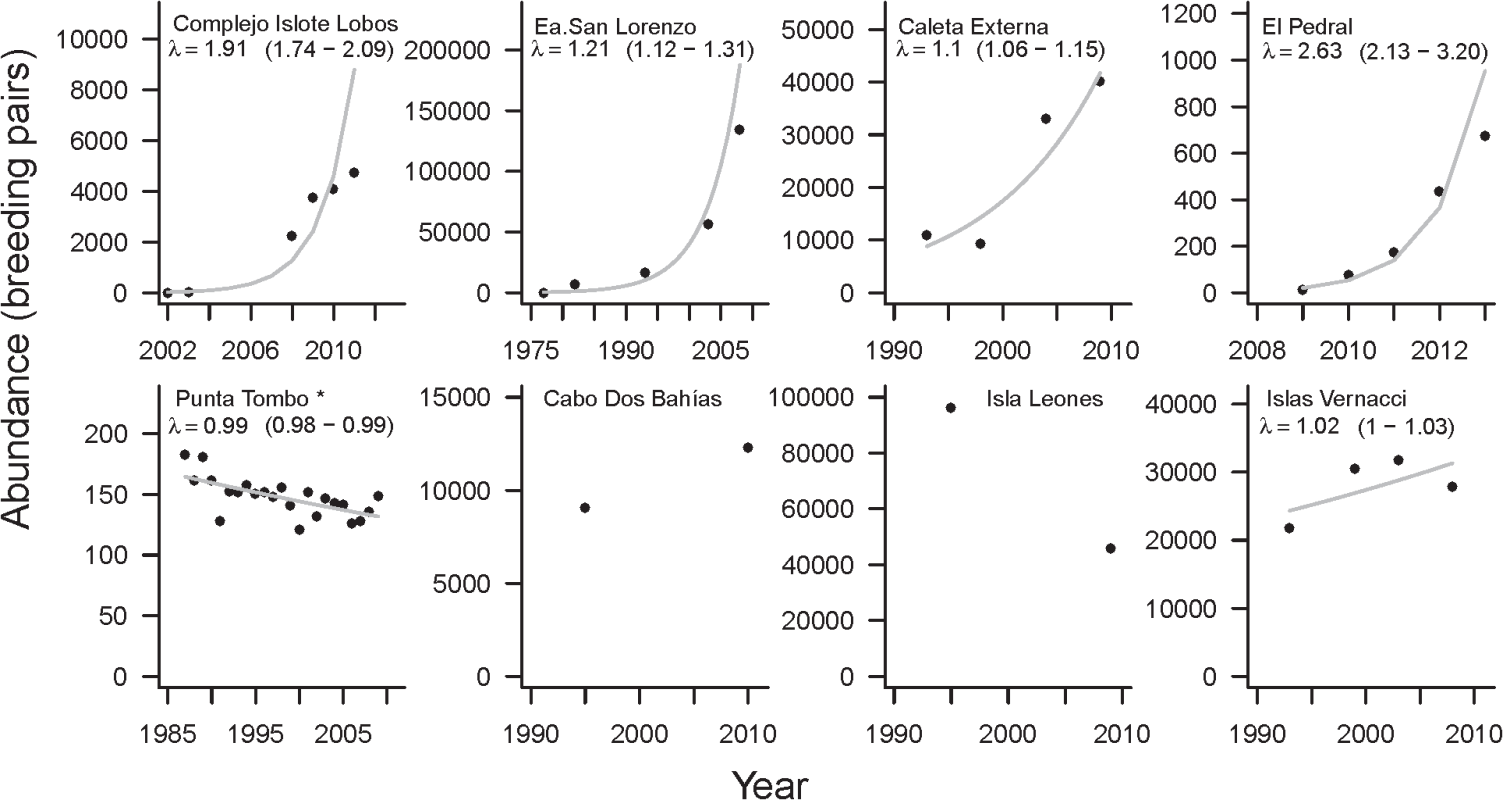
Abundance data and growth rates for 8 Magellanic penguin colonies. Northern colonies (above) and southern colonies (below). Abundance data (2008–2012) are from this study. Other estimations of colony size are from the literature [40,41,43,44,52,93,94] and Yorio and Garcia Borboroglu unpublished data (Islas Vernacci 1999). *For Punta Tombo, data correspond to an index of abundance. Finite rate of increase (*λ*) is provided for colonies with at least four data points. Values in brackets correspond to the confidence interval of the rate.

We updated information on colony size (number of active nests or breeding pairs) in all but one colony (Punta Tombo) between 2008 and 2013 and integrated these data with previously published information to assess population trends (see Fig. 2 for details). At Punta Tombo, we used counts of the number of active nests in 19, 100m^2^ permanent plots spaced 100 meters apart through the colony each year in October since 1987 [15]. The sum across all plots provides an index of abundance that was treated similarly to the rest of the colonies. We did surveys after the time of peak egg laying (maximum occupation of nests in the colony) [48].

We estimated population trends using the exponential, density-independent, discrete-time model [49]: *N_t_* = *N_to_* *λ^(t−to)^* where *N_t_* is the estimated population size in the last year, *N_to_* is the population size during an initial year *t*_*o*_, *λ* is the finite rate of increase and *t* is the time interval of the estimations (one year). A population is stable when *λ* = 1, so that *λ* − 1 indicates the magnitude of the increase (*λ* > 1) or decline (*λ* < 1) respectively. For practical purposes, we used a natural log transformation of population size as the dependent variable in a linear regression model with time as an independent variable. The slope provides an estimate of the instantaneous rate of increase (r), which relates to *λ* through *λ* = *e^r^*. We estimated confidence intervals for the trend by bootstrapping the residuals of the linear regression [50] in all colonies with at least four data points. The estimation method assumed a deterministic population growth model and attributed all variability in the data to errors in the estimates of population size (i.e. observation error model) [51].

Given that for Cabo Dos Bahías and Isla Leones we only have two data points, these colonies were excluded from all analyses regarding growth rates and they are only presented in the figure (Fig. 2) showing the data points available that supported our interpretation on population trends.

To evaluate the relationship between the estimated growth rate with population size and the estimated age of colonies, we performed linear regressions between the instantaneous growth rate (r) and the natural logarithm of the corresponding independent variables, a transformation that allowed us to make the relationship linear. We estimated age as the difference between year 2013 and the year of first report in the literature: Complejo Islote Lobos, 11 years [41]; Ea. San Lorenzo, 36 years [52]; El Pedral, 4 years (this study); Punta Tombo 93 years [53] and Islas Vernacci, 199 years [54]. For Caleta Externa, we assumed the same age as Ea. San Lorenzo.

### Productivity of colonies

We measured breeding success and assessed body condition of chicks close to the time of fledging in all colonies during at least two different years: in 2008, colonies Complejo Islote Lobos, Ea. San Lorenzo, Punta Tombo, Cabo Dos Bahías and Islas Vernacci for both breeding success and body condition of chicks. In 2009, Complejo Islote Lobos, Caleta Externa, El Pedral, Punta Tombo and Isla Leones (both breeding success and body condition) and Cabo Dos Bahias (body condition). In 2010, all colonies were visited again but in El Pedral, due to total breeding failure, body condition of chicks could not be evaluated. During 2011, we measured both breeding success and body condition in this colony. Breeding success was defined as the number of chicks fledged per nest with at least one egg present in October for each breeding season. We measured breeding success within 100 m2 circular plots spaced at least 20m apart within each colony, which were checked twice during the breeding season. We counted the number of nests with at *least* one egg in October at the time of peak egg laying, and counted the number of chicks in the same plots a second time before they fledged during January. At Pedral and Punta Tombo breeding success was calculated by the success of individual nests.

When we found differences in the mean breeding success between large sectors within our study area, we compared them by calculating the confidence interval of the difference between means with a bootstrap [50].

During the second nest check in late January, we randomly selected 30 chicks in all colonies except at Islas Vernacci (where we selected 23 chicks during 2010). We weighted them using a 5 Kg spring scale and measured flipper length with a ruler. We calculated a body condition index by dividing body mass with flipper length [55,56]. To evaluate the relationship between body condition of chicks and colony size, we used general linear mixed effect models (GLMM) with colony size as a fixed factor and year and colony identity as independent random effects, as follows:

Body condition ∼ intercept + colony size + (1 | season) + (1 | colony ID)

### Foraging distance

We deployed 24 satellite transmitters (*Platform Transmitter Terminals*, PTT) on Magellanic penguins breeding at Complejo Islote Lobos (2010), El Pedral (2011 and 2012) and Isla Leones (2009 and 2010). All satellite tags were attached between mid-December and mid-January, when adults were feeding chicks that were > 30 days old following Boersma et al (2009). We received positions via Argos satellites (Service Argos, Largo, Mariland, USA). For accuracy, we used only 1–3 Argos location classes, in which the transmitters received at least four messages. We plotted satellite tracks in QGis v 1.7.4 [57] and removed individual positions that were clearly far outside of the track (error positions). We calculated trip distances following Boersma et al. (2009) and averaged all trips from penguins in each colony to calculate mean distances. These data were integrated with published information from other colonies during the late chick rearing period: San Lorenzo (∼La Ernestina, 91Km), Punta Tombo (134 Km) and Cabo Dos Bahías (67 Km) [20] to evaluate the relationship between mean trip distances and colony size by linear regression.

### Comparing projection from closed population models to observed trends

To assess the potential growth of colonies due to their internal dynamics, we estimated the finite rate of increase as the dominant eigenvalue of a post-breeding transition matrix (*Leslie matrix)* [58] for each individual population, and contrasted them with those from abundance data.

Maximum age of Magellanic penguins exceeds 30 years of age [42]. Consequently, we used matrices with thirty five age classes (with no accumulation class).

Fecundity per age class (*F_a,c_*) in each individual colony was calculated as:

$$F_{a,c}=m_{a}*\frac{BS_{c}}{2}$$

*m_a_* is the proportion of mature females at age class *a*. Boersma et al. (2013) reported about 50% of females mature at age 6, so we used *m*_6_ = 0.5 and considered older age classes to be mature (m = 1).

*BS_c_* is the mean breeding success for each individual colony, which was divided by 1/2 given that the transition matrix only included females. We used our own data and included published information for Ea. San Lorenzo (years 2007 and 2009) [59], Punta Tombo (1983–2006) [15] and Islas Vernacci (1999–2000) [60].

Adult survival was estimated at 0.87 for web-tagged adult breeding Magellanic penguins [61] but juvenile (one–year- old) survival is unknown. For the closely related African penguin (*Spheniscus demersus*), estimates range from 0.10 (CI 0.06–0.15) to 0.53 (0.43–0.62) [62]. Because our aim was to evaluate if the observed growth rates could be attainable in the absence of dispersal, we arbitrarily increased point estimates, using 0.65 for juvenile survival and 0.87, 0.91 and 0.95 for adults (older than one year old). In any case, we anticipated that analysis would be conservative.

We contrasted the ensuing range of finite rate of increase derived from the matrices (considered to be a measure of maximum potential growth) with the observed population growth in colonies to explore if the observed trends could be attained if populations were closed.

In all data analysis, we used R v 3.0.2 [63] and the libraries gplots [64], chron [65], rgdal [66], popbio [67] and lme4 [68].

## Results

### Population trends

Mean population growth rates ranged from a minimum of 0.99, which indicates a declining colony at a 1% annual rate, to a maximum of 2.63 which represents a colony more than doubling its size every year (Table 1, Fig. 2). Although the data are not adequate to formally test explanations for the differences in colony trends, we found some clear differences between colonies located in the northern part of our study area (herein “northern sector”) and those located in the southern part of our study area (herein “southern sector”). Colonies located in the northern sector had consistently high growth rates whereas the growth of colonies located in the southern sector depended on their sizes, with smaller populations remaining relatively stable and larger colonies declining.

The growth rate was inversely related to both log colony size (R^2^ = 0.84, *F*_1,4_ = 21.66, *β* = −0.17, 95% CI [−0.27,−0.07]) and to the log estimated age of the colony (R^2^ = 0.88, *F*_1,4_ = 29.58, *β* = −0.26, 95% CI [−0.4, −0.13])

**Table 1:** Summary data for 8 Magellanic penguin colonies in Northern Patagonia.

Colony	Age	Population size	Growth rate	Mean breeding success	Mean body condition	Mean Foraging distance
Complejo Islote Lobos (Cil)	11	4748(2011)	1.91(6)	1.27(0.11)	20.68 (2.92)	25.26(10.36)
Ea. San Lorenzo (Slor)	36	134416(2008)	1.21(5)	1.15(0.32)	18.35 (2.64)	91 (18)^b^
Caleta Externa (Cal)	36	40225(2009)	1.10(4)	1.12(0.01)	18.33 (4.83)	
El Pedral (Ped)	4	675(2013)	2.63(5)	0.82(0.63)	21.89 (2.23)	15.69(6.39)
Punta Tombo (Tom)	93	200000(2006^a^)	0.99(23)	0.47(0.2)	17.73 (3.77)	134(16)^b^
Cabo Dos Bahías (C2b)		12295(2010)		0.95(0.76)	20.56 (2.89)	67(22)^b^
Isla Leones (Leo)		45842(2009)		0.15(0.16)	20.13 (2.97)	44.49(62.01)
Islas Vernacci (Vern)	199	27895(2008)	1.02(4)	0.72(0.36)	18.97 (3.49)	

The estimated age of colonies was taken as the difference between 2013 and the first year of report in the literature: Cil [41], Slor [52], Ped (this study), Tom [53], Vern [54]. For Cal, age was assumed the same as Slor. Numbers in parenthesis indicate: the year of the last estimate of population size, the number of years used for the growth rate estimation and standard deviation of breeding success, body condition and foraging distance respectively. Mean breeding success (BS) and body condition (BC) correspond to years 2008 to 2010 in Cil and Tom (BS and BC); 2008 and 2010 in Slor and Vern (BS and BC) and C2b (BS); in 2008 to 2010 C2b (BC) and 2009 and 2010 in Cal and Leo (BS and BC). In Ped, BS included years 2009 to 2012 while BC included years 2009 and 2011.

^a^Population size taken from [15].

^b^Data from [20].

### Productivity of colonies and foraging distance

**Fig 3:**
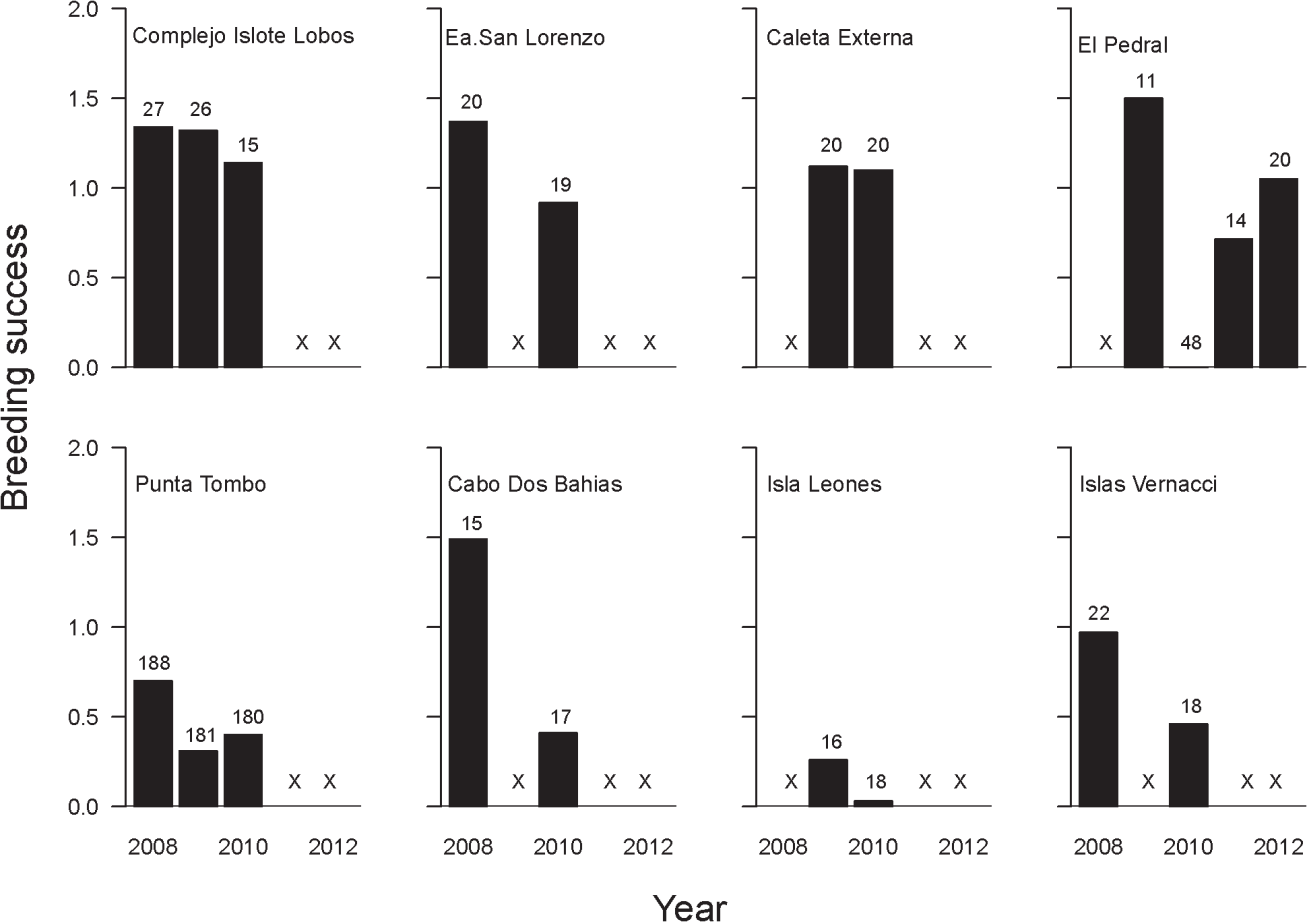
Annual breeding success for 8 Magellanic penguin colonies. Northern colonies (above) and southern colonies (below). X indicates no data for that year. Numbers above bars indicate n (number of circular plots or nests).

Mean breeding success was higher in the northern sector (Table 1, Fig. 3) (mean BS = 1.05, SD = 0.41, n = 11) than in the southern sector (mean BS 0.56, SD = 0.44, n = 9) (CI for the difference between means = 0.10–0.82). Breeding success in the northern sector was even higher (mean BS = 1.19, SD = 0.16, n = 7) when excluding El Pedral colony (CI for the difference between means = 0.29–0.88), where an anomalous year without reproduction occurred in 2010, likely due to demographic stochasticity in this very small colony.

**Fig 4:**
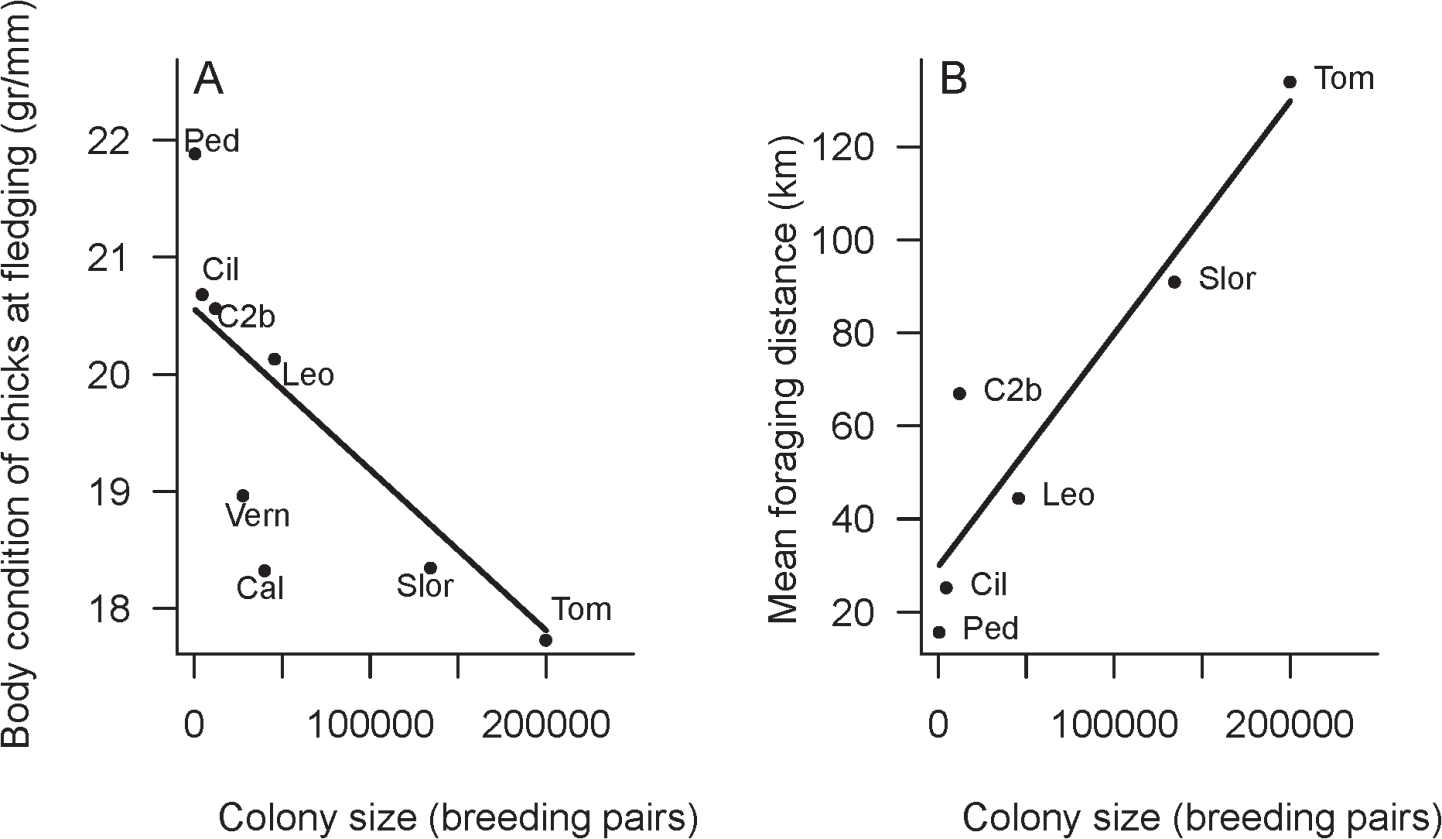
A) Body condition of chicks at fledging and colony size. Points indicate mean body condition (from at least two different years). Fitted line corresponds to a general mixed effect model (GLMM) with colony size as a fixed factor, and years and colony ID as independent random effects. Labels indicate the name of the colonies: Cil (Complejo Islote Lobos), Ped (El Pedral), Slor (Ea. San Lorenzo), Cal (Caleta Externa), Tom (Punta Tombo), C2b (Cabo Dos Bahías), Leo (Isla Leones), Vern (Islas Vernacci). Colony size for Tom is from [15]. **B) Foraging trip distance and colony size**. Labels indicate the name of the colonies: Cil (Complejo Islote Lobos), Ped (El Pedral), Slor (Ea. San Lorenzo), Tom (Punta Tombo), C2b (Cabo Dos Bahías), Leo (Isla Leones). Colony size for Tom is from [15]. Mean trip distances for Slor, Tom and C2b are from [20].

Body condition of chicks was inversely related to colony size (*β* = −1.37e^−5^, 95% CI [−2.29e^−5^, −5.52e^−6^], Fig. 4A) and mean foraging trip distances increased linearly with colony size (R^2^ = 0.86, *F*_1,4_ = 25.42, *β* = 5 e^−4^, 95% CI [2.22 e^−4^,7.76 e^−4^], Fig. 4B)

### Comparing projections from closed-population models to observed trends

**Fig 5:**
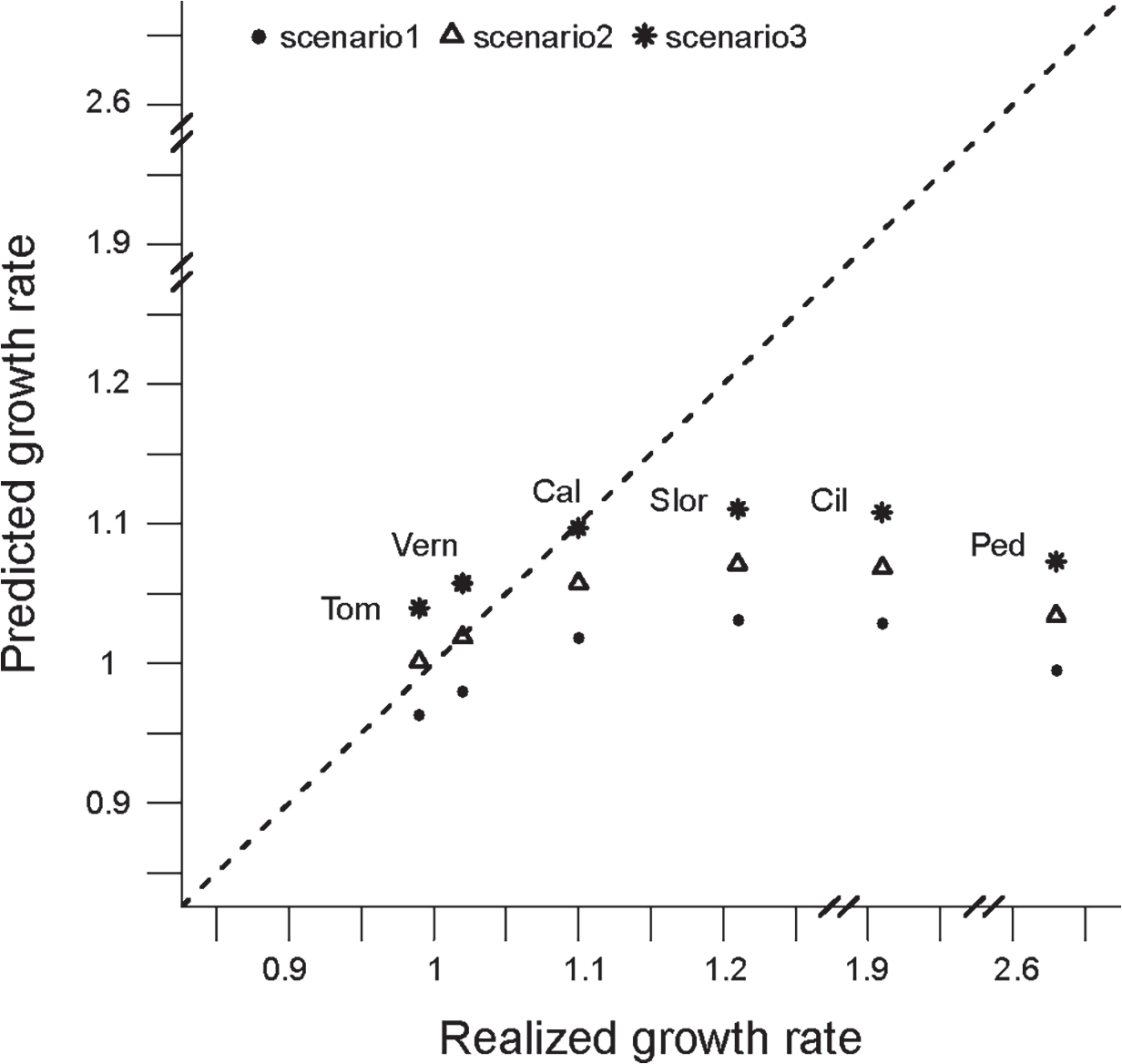
Comparison between the realized growth rates (obtained from abundance data) and estimated from modeling. Labels indicate the name of the colonies: Cil (Complejo Islote Lobos), Ped (El Pedral), Slor (Ea. San Lorenzo), Tom (Punta Tombo), Vern (Islas Vernacci). Points, triangles and asterisks indicate scenarios (combinations of juvenile and adult survival used to model the predicted growth rates based on transition matrices).

In the northern sector, colony growth was higher than what their internal dynamics predicted (Fig. 5). The maximum potential growth rates from transition matrices were lower (Range _CIL_ = 1.029–1.108; Range _SLOR_ = 1.031–1.111; Range _CAL_ = 1.018–1.097; Range _PED_ = 0.995–1.073) than those observed (*λ_CIL_* = 1.91, *λ_SLOR_* = 1.21, *λ_CAL_* = 1.10, *λ_PED_* = 2.63, described above, Fig. 5) for all combination of survival parameters. For the colonies evaluated in the southern sector, all realized rates fell into the ranges of maximum potential growth rate (Range _TOM_ = 0.963–1.040; Range _VERN_ = 0.980–1.057), indicating that the observed growth (*λ_TOM_* = 0.99, *λ_VERN_* = 1.02) could be explained by their intrinsic productivity and survival.

## Discussion

Magellanic penguin colonies scattered along the Atlantic northern distribution varied in their growth patterns. Younger colonies established during the last decades in the northern sector, increased at high rates, with the recently settled, smallest populations growing the fastest. By contrast, in the southern Chubut sector, where colonies were long-established, the large breeding aggregations declined while smaller populations remained relatively stable. Some colonies (Cabo Dos Bahias and Isla Leones) have few data points, which precludes the estimation of lambda values. However, additional information suggests a pattern of stability and a substantial decline for both colonies respectively. Despite of an increase in the number of breeding pairs (which may be attributable to residual variance) at Cabo Dos Bahias, the pattern of nest occupancy and colony area do not suggest a substantial change during the last 15 years. By contrast, the number of active nests at Isla Leones colony has been reduced to half of the abundance previously reported [43].

Growing colonies had higher breeding success, but their internal productivity could not account for their high growth rates. The comparison among growth rates estimated from abundance data and those predicted from population modeling [58] indicated that immigration played a major role in the steady increase of northern colonies. The recent establishment of Pedral and Complejo Islote Lobos also highlighted the influential role of dispersal in local population dynamics. Limited genetic structuring among colonies that depend on similar foraging grounds suggests that source populations might be within the same oceanographic region [46]. Therefore, the decline of the largest colonies of the southern sector, Punta Tombo and Isla Leones, could have been due in part to the emigration of penguins that recruited in the northernmost area. These colonies were among the most abundant during the 90's, accounting for more than 25% of the Argentine population [40,41,43].

The factors and mechanisms driving the metapopulation in Northern Patagonia are difficult to disentangle. However, life history traits in seabirds predict that natal rather than breeding dispersal is the most common mechanism underlying their movement [69], due to the high breeding fidelity for most species [70]. Several studies have shown that seabird metapopulations are sustained by the recruitment of young in non natal colonies [71,72,73]. Given that Magellanic penguins are faithful to their colonies once they start breeding [15,42], the recruitment of young in non natal colonies might be expected, probably attracted by the higher performance of the northern colonies. Recruitment processes in seabirds are still poorly understood [4,70], but young birds of many species often rely on breeding success of the colonies as an indicator of environmental quality [8,38]. Provided the long distances Magellanic penguins migrate northward after the breeding season[42,74,75], young penguins may have several opportunities to prospect different colonies in their way back from wintering areas. Prospection may also occur during the breeding season, at the times when juveniles and young adults concentrate on beaches [76] and can gather information of the quality of the colonies.

The differences in the productivity of colonies we report are likely result of favorable environmental conditions in the north and probably, density dependent processes, exacerbated by environmental variability in the southern sector. The Northern Patagonia Frontal System, a tidal front that extends from 41°—42° to 45°S, is the most conspicuous oceanographic feature in Northern Patagonia [77,78]. It promotes favorable conditions for fish spawning, particularly in the north, at the latitude of Península Valdés [79], where both richness and biomass of pelagic fish are higher [80]. By contrast, colonies in the southern sector lie within the southern end of the tidal front, where conditions tend to be more variable [20,45,47], likely generating a more variable productivity. Oceanographic conditions are an important driver in the dynamics of Magellanic penguins [20] and at Punta Tombo for example, foraging distance determined breeding success [81].

Despite the differences in environmental conditions between sectors [20,45,47], density independent factors alone do not seem sufficient to explain the overall pattern we found. Several pieces of information suggest that population abundance may have influenced the productivity of colonies as well. Our results, despite the few data, suggest a pattern of density dependent growth, which predicts an inverse relationship between the annual growth rates and population size [82].

Several studies suggested that, as a consequence of density dependent mechanisms operating at sea, seabirds breeding in larger colonies show reduced breeding performance, either in terms of breeding success and/or chick growth [33,34]. We found that both foraging distance increased with colony size and that chicks in better body condition from the smallest colonies, regardless of their location. Given that Magellanic penguins remove increasingly larger biomass of prey throughout the breeding period, density dependent mechanisms may operate at short time scales [83,84]. As chicks grow larger and their foraging demands increase, both parents start foraging simultaneously, at farther distances than in the previous stage when both members of the pair do not forage simultaneously [81]. The higher food requirements and the need to forage close to the colony to reduce travel time, coupled with the higher number of individuals foraging at the same time may reduce per capita food intake in large colonies.

Density dependence operates strongly in years of low resource availability[25,85]; hence, the effect of population numbers might be particularly important in the southern sector, where environmental conditions tend to be more variable. Long term studies at Punta Tombo have shown that, over a period of 24 years, breeding success never exceeds the value of one [15]. By contrast, breeding success at the comparatively large colony located in the northern sector (Ea. San Lorenzo), exceeds this value in just one out of the two years in which we collected data, been the other equally high (0.92). Additional information for the same colony also showed high productivity in terms of the number of chicks fledged in other years [59]. All these facts suggest that the effect of population numbers may be exacerbated by environmental conditions. In this sense, the favorable conditions and comparatively, more stable conditions in the northern sector, likely counteract the effect of foraging distance in the larger colonies of this sector. The considerable short time that penguins breeding in Peninsula Valdes spend foraging suggests that the frontal area offers high prey availability [45].

Previous studies attributed all differences in the dynamics among colonies to environmental conditions [20,45]. However, those studies involved colonies from distant regions with completely different features, some of them located south of 45°S [20,45]. Given that contrasting differences in oceanographic conditions north and south of 45°S determine distinct foraging grounds [20,45,47], as well as different prey types for Magellanic penguins [47,86,87], differences in the dynamics of colonies from Northern and Southern Patagonia may also be expected.

Our results suggest that a complex interaction between density dependence and environmental factors may operate in penguin population dynamics. Even though few studies have documented the influence of both factors taking place simultaneously in marine systems [88,89], this model is fairly typical of other vertebrate populations, with vital rates regulated by environmental factors through the supply of food, such as red kangaroos [90] or large ruminants [91] responding to rainfall. In Magellanic penguins, the combination of both factors operating simultaneously might have been responsible for the lower performance in the -once- largest colonies of the southern sector. Given that prospecting seabirds often rely on breeding success as a cues of environmental quality[37,38,39], these factors may have promoted the recruitment of penguins in the northern area, contributing to the observed increase of these colonies.

Our study provides the first attempt at understanding the dynamics of Magellanic penguin populations at a wide geographical scale. The prominent role that immigration played in the steady increase of northern colonies highlights the need to consider population connectivity, scaling up the spatial perspective to properly understand population changes. Dispersal still remains one of the major gaps in seabird research [4]. Large-scale banding and resighting programs, not only are logistical and economically difficult, but they also tend to underestimate movement rates [92]. Consequently, seabird colonies are often studied as isolated units, with the implicit assumption that dispersal has a negligible effect on local population dynamics. As a result, growth rates are assumed to reflect only the internal demographic processes influencing abundance, natality and mortality.

Within a context of population connectivity, however, the internal demography is confounded with dispersal and the population behavior at the colony level can only be fully understood by understanding their links with neighboring colonies. In the end, ignoring the spatial structure in a context of population connectivity may lead to wrong conclusions about the underlying mechanism driving demographic changes, the status and value of different colonies for the species well-being, or the value of particular conservation policies. We suggest that, in light of the increasing evidence of seabird colonies behaving as a metapopulation [5,6,8,46], the scale for population assessments and viability analysis should be re-considered.
